# Emergency Medicine Faculty Utilization of Point-of-Care Ultrasound in the Clinical Setting

**DOI:** 10.7759/cureus.78868

**Published:** 2025-02-11

**Authors:** Frances M Russell, Robinson M Ferre, Sarah K Kennedy, Benjamin Nti, Drew Frey, Daniel Brenner

**Affiliations:** 1 Emergency Medicine, Indiana University School of Medicine, Indianapolis, USA; 2 Pediatric Emergency Medicine, Indiana University Health, Indianapolis, USA; 3 Emergency Medicine, Southern Illinois University School of Medicine, Springfield, USA

**Keywords:** clinical usage, emergency medicine physician, faculty, point-of-care-ultrasound, utilization

## Abstract

Introduction

Point-of-care ultrasound (POCUS) curricula for emergency medicine residents and faculty are guided by emergency medicine societal ultrasound guidelines. These guidelines lack clinical data to support them and are mainly based on expert consensus recommendations. Data are needed to address critical gaps in the literature to identify which POCUS studies are most commonly utilized in the clinical setting to help guide guideline recommendations and POCUS curricular design. The primary aim of this study was to determine the most utilized POCUS modalities in the emergency department clinical setting. The findings of this study may be used to guide the curricular design of future POCUS trainings.

Methods

This was a retrospective study evaluating all clinically indicated and billed POCUS studies performed and interpreted by faculty in the emergency department setting across 10 emergency departments over a three-year period in Indianapolis, Indina, USA. The number of exams and modalities were extracted from the POCUS workflow solution. The frequency and percentage of exams were calculated.

Results

A total of 5,324 POCUS examinations were performed. Cardiac, obstetric, soft tissue, and focused assessment with sonography in trauma (FAST) POCUS were the most billed modalities across all adult emergency departments regardless of academic or community setting. Although fewer data were available from the pediatric setting, we found that cardiac, soft tissue, FAST, and lung POCUS exams were the most utilized.

Conclusion

These data from a single healthcare system would suggest that emergency physician POCUS curricula should focus on cardiac, obstetric, soft tissue, FAST, and lung exams. More data are needed from the pediatric setting to determine which scans are most utilized.

## Introduction

Point-of-care ultrasound (POCUS) is widely used in the emergency department to augment patient care by narrowing down a differential diagnosis, guiding treatment and procedures, and risk-stratifying patients [1-6]. Guidelines for POCUS utilization published by the American College of Emergency Physicians date back to 2001 and continue to update as POCUS continues to expand within the field of emergency medicine [1]. For a decade, POCUS has been a core competency of emergency medicine residency training [7], and more recent literature has focused on POCUS training for emergency physicians who did not learn POCUS during their training [8-10].

POCUS training curricula for residents and post-residency physicians are created from the American College of Emergency Physicians (ACEP) ultrasound guidelines [1], which are largely based on expert consensus and not based on clinical data. These guidelines have emphasized focused assessment with sonography in trauma (FAST), first trimester obstetric, cardiac, and aorta examinations, but it remains unknown whether these exams are the most frequently used in the clinical setting and if these exams should continue to be the emphasis of POCUS training. In addition, very little data exists regarding pediatric and community faculty usage of POCUS [10,11]. There remains a critical gap in the literature addressing which POCUS exams are being integrated into clinical decision-making. These data are needed to inform emergency medicine residency POCUS curricula development and continuing medical education POCUS curricula development for post-residency emergency physicians learning POCUS as these are the exams that will likely be utilized after training. The primary aim of this paper was to determine which POCUS modalities are most utilized in the emergency department clinical setting. The secondary aim was to evaluate for differences in utilization patterns based on practice setting, i.e., community, academic, adult, and pediatric, to evaluate if practice setting impacts which POCUS exams are used.

## Materials and methods

Study design and setting

This was a retrospective study on clinically indicated POCUS examinations performed by emergency medicine faculty in the emergency department clinical setting over a three-year span from January 1, 2019, to December 31, 2021. This study was approved by the Indiana University Institutional Review Board (IRB) (approval no. 16102) and deemed exempt with a waiver of informed consent.

Faculty were from three large urban academic hospitals (Methodist Hopsital, Eskenazi Hospital, Riley Children’s Hospital, Indianapolis, IN): two adult emergency departments with a combined annual volume of >180,000 patient visits per year, one pediatric emergency department with around 60,000 patient visits per year, and seven community sites with volumes ranging from 6,000 to 55,000 patients per year. All faculty were trained in POCUS through the residency or practice pathway as outlined by the ACEP guidelines [1]. All members of the ultrasound division, including adult and pediatric emergency medicine faculty, have a focused practice designation (FDP) in Advanced Emergency Medicine Ultrasonography, completed ultrasound fellowship training, or met the criteria to sit for the FDP examination.

All POCUS examinations performed by a credentialed emergency medicine faculty that were billed and sent to the electronic medical record were included. Exams were completed by faculty alone or by a resident or student with faculty supervision. Education-only POCUS exams, non-billed exams, and exams without patient information were excluded. We also excluded procedural ultrasounds as documentation of these exams was not commonly performed during this time period and billing data for POCUS-guided procedures was captured through the electronic medical record and not QPath. The number of POCUS exams and type of exam were extracted from the POCUS workflow solution (QPath, Telexy) by students and ultrasound fellows who were blinded to the study outcome. Billed exams were identified using QPath. Students and fellows were trained by a faculty member on data extraction with routine monitoring occurring weekly. Only billed exams were included as to be billed the exam had to be reviewed and signed by an emergency medicine faculty credentialed in POCUS and the exam was used to guide patient care, meaning that the exam had to be clinically indicated. Educational exams are commonly performed by residents and medical students in the clinical setting, but do not have any clinical impact so were excluded. 

Statistical analysis

Data were exported from QPath into Microsoft Excel (Microsoft, Redmond, WA). Statistical analysis was done using Microsoft Excel and VassarStats (http://vassarstats.net Poughkeepsie, NY), and the frequency and percentages of exams were reported.

## Results

Over a three-year span within a single healthcare system, 5,324 POCUS examinations were performed: 1,345 in 2019, 1,851 in 2020, and 2,128 in 2021 (Table 1). When excluding the exams performed by ultrasound division faculty members, 2,401 POCUS studies were completed over three years. Most POCUS examinations were completed in the adult academic setting compared to community and pediatric emergency department settings.

**Table 1:** Point-of-care ultrasounds performed by site with and without ultrasound division faculty

Year		Academic	Community	Pediatric	Total
2019	Number of faculty	33	1	9	43
	Number of billed scans	1210	4	131	1345
2020	Number of faculty	42	11	8	61
	Number of billed scans	1565	173	107	1845
2021	Number of faculty	40	16	8	64
	Number of billed scans	1681	347	96	2124
Total billed scans		4456	524	334	5314
Without US Division		Academic	Community	Pediatric	Total
2019	Number of faculty	24	1	8	33
	Number of billed scans	401	4	40	445
2020	Number of faculty	33	11	7	51
	Number of billed scans	697	173	87	957
2021	Number of faculty	29	16	7	52
	Number of billed scans	593	347	51	991
Total billed scans		1691	524	178	2393

**Figure 1:**
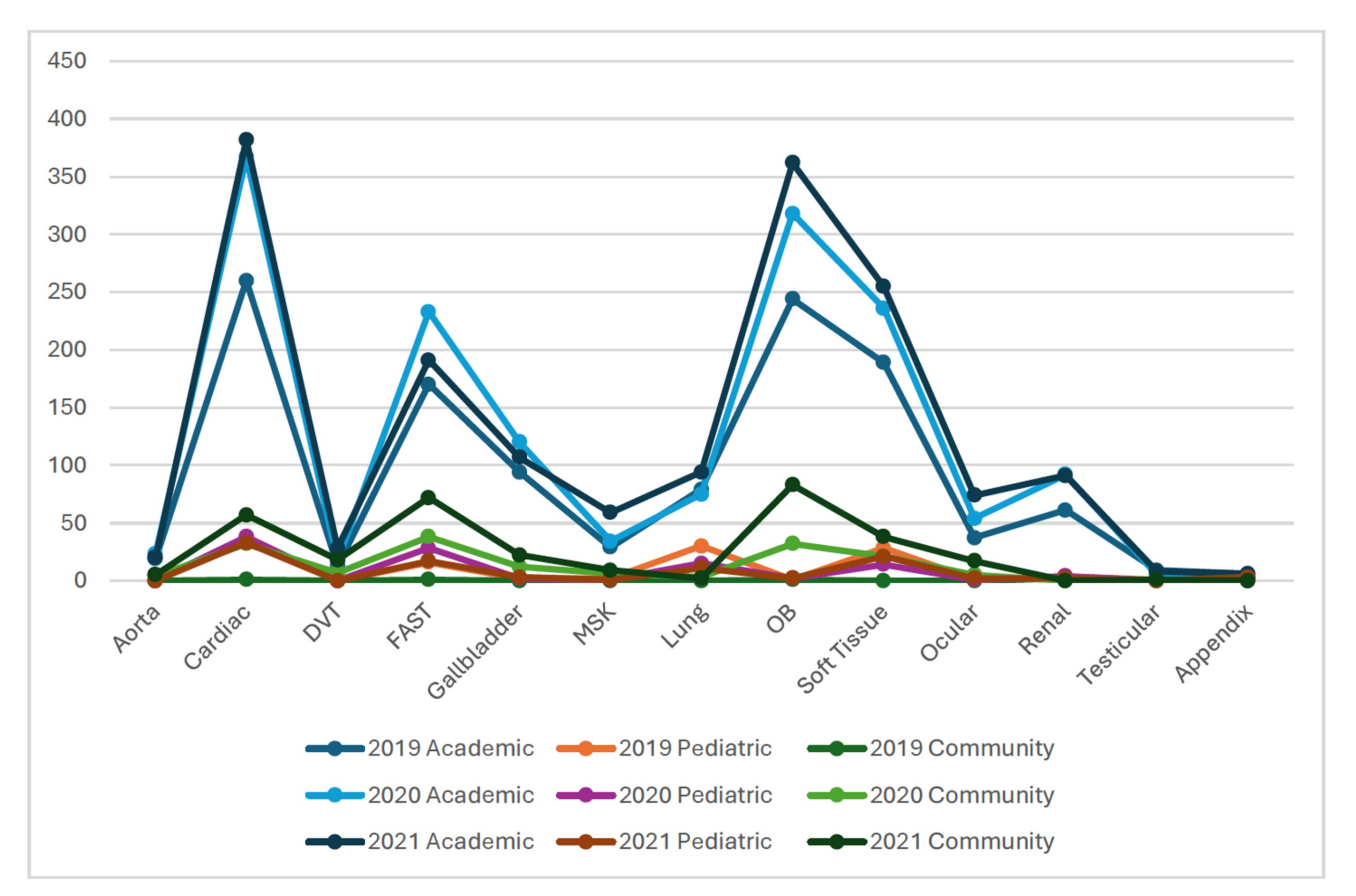
Point-of-care ultrasound (POCUS) examinations by number performed, modality, and clinical setting DVT: deep venous thrombosis, MSK: musculoskeletal, OB: obstetric

The most frequently billed scans were cardiac, obstetric, soft tissue, and focused assessment with sonography in trauma (FAST) across all adult departments regardless of academic or community setting (Table 2). These were also the most frequently billed scans when excluding those performed by ultrasound division faculty. In the pediatric setting, cardiac, soft tissue, FAST, and lung exams were the most utilized POCUS examinations when including and excluding ultrasound division faculty. In the academic setting gallbladder, lung, renal, and ocular exams were also frequently performed, while aorta and deep venous thrombosis (DVT) were less commonly performed, and testicular, appendix, and nerve block exams were rarely performed. In the community setting gallbladder, DVT and ocular were moderately performed, while renal and lung exams were rarely performed. In the pediatric setting, appendix, musculoskeletal, and ocular exams were rarely performed (see Figure 1).

**Table 2:** Number of billed point-of-care ultrasound examinations by modality and site over three years US: ultrasound, OB: obstetric, FAST: focused assessment with sonography in trauma, MSK: musculoskeletal, DVT: deep venous thrombosis

	Academic n, %		Academic w/o US division n, %		Pediatric n, %		Pediatric w/o US division n, %		Community n,%	
Cardiac	1009	24.28	273	16.07	108	33.13	69	54.76	90	18.44
OB	924	22.24	588	34.61	5	1.53	2	1.59	116	23.77
Soft tissue	680	16.37	257	15.13	63	19.33	20	15.87	59	12.09
FAST	594	14.30	309	18.19	61	18.71	14	11.11	111	22.75
Gallbladder	321	7.19	54	3.18	7	2.15	3	2.38	34	6.97
Lung	248	5.97	33	1.94	56	17.18	12	9.52	5	1.02
Renal	244	5.87	71	4.18	10	3.07	4	3.17	1	0.20
Ocular	165	3.97	61	3.59	4	1.23	0	0	22	4.51
MSK	122	2.94	21	1.24	4	1.23	0	0	15	3.07
Aorta	62	1.49	29	1.71	0	0	0	0	9	1.84
DVT	58	1.40	1	0.06	0	0	0	0	25	5.12
Testicular	22	0.53	0	0	1	0.31	0	0	1	0.20
Appendix	15	0.36	1	0.06	7	2.15	2	1.59	0	0

Table 3 shows the breakdown in the number of billed POCUS examinations by setting, year, and exam type.

**Table 3:** Point-of-care examinations by setting, year and exam type. OB: obstetric, FAST: focused assessment with sonography in trauma, MSK: musculoskeletal, DVT: deep venous thrombosis

Year	Exam	Academic		Pediatric		Community
		All	Without division	All	Without division	
2019	Aorta	19	9	0	0	0
	Cardiac	260	51	37	12	1
	DVT	13	0	0	0	0
	FAST	170	87	16	3	1
	Gallbladder	94	12	2	0	0
	MSK	29	0	2	1	0
	Lung	79	7	30	4	0
	OB	244	156	1	0	1
	Soft tissue	189	59	28	8	0
	Ocular	37	7	1	0	0
	Renal	61	13	4	0	1
	Testicular	9	0	1	0	0
	Appendix	6	0	2	0	0
2020	Aorta	23	11	0	0	4
	Cardiac	367	127	38	32	32
	DVT	17	0	0	0	7
	FAST	233	144	28	5	38
	Gallbladder	120	21	2	2	12
	MSK	34	7	1	1	6
	Lung	75	22	15	9	3
	OB	318	212	2	2	32
	Soft Tissue	236	99	14	5	21
	Ocular	54	24	1	1	5
	Renal	92	29	4	4	0
	Testicular	5	0	0	0	0
	Appendix	3	1	2	0	0
2021	Aorta	20	9	0	0	5
	Cardiac	382	95	33	25	57
	DVT	28	1	0	0	18
	FAST	191	78	17	4	72
	Gallbladder	107	21	3	2	22
	MSK	59	14	1	0	9
	Lung	94	4	11	1	2
	OB	362	220	2	1	83
	Soft Tissue	255	99	21	7	38
	Ocular	74	30	2	1	17
	Renal	91	29	2	0	0
	Testicular	8	0	0	0	1
	Appendix	6	0	3	0	0

Significantly fewer faculty were billing for POCUS examinations in the community and pediatric setting when compared to the academic setting. In the academic setting, there were an average of 38 faculty over the three-year billing for POCUS compared to an average of nine in the community setting and eight in the pediatric setting. A greater proportion of the total faculty in the academic adult and pediatric setting were billing for POCUS scans as well; 68% (50/74)of the academic faculty billed for at least one POCUS scan, while in the community, only 40% (16/40) of the faculty billed for at least one POCUS scan. Adult academic faculty had a higher average number of scans performed per faculty (average 39 scans) than community (average 19 scans) or pediatric faculty (average 13 scans).

## Discussion

While guidelines are in place to help guide POCUS training curricula for emergency medicine residents and faculty [1], they are based largely on expert opinion due to a lack of clinical data to inform guidelines and recommendations. To generate an evidence base for these recommendations, this study evaluated the usage of POCUS examinations being performed for clinical care by trained faculty in different practice settings across ten emergency departments within one healthcare system. Across all clinical settings and with all users, the most billed POCUS exams were cardiac, FAST, and soft tissue. In the community and academic setting obstetric POCUS was highly utilized and in the pediatric setting lung POCUS was highly utilized.

The utilization patterns observed in this study are likely multifactorial. The first and most likely factor is the lack of ability to immediately obtain alternative imaging, specifically for cardiac and FAST exams, which are commonly performed at the bedside in hemodynamically unstable patients. Being able to use POCUS quickly during a patient’s evaluation can help guide diagnosis and management decisions [12]. Soft tissue and lung ultrasounds were frequently used by physicians in pediatric and academic settings. Factors contributing to the higher use of these exams include the relative ease in learning how to perform and interpret them compared to other POCUS modalities. In addition, both soft tissue and lung exams can significantly impact clinical decision-making [13,14]. In the pediatric setting, using POCUS to evaluate skin and soft tissue infections and patients with dyspnea can significantly decrease radiation exposure, emergency department length of stay, and healthcare costs [15,16]. The high use of obstetric ultrasound in the community and academic setting was likely a result of long wait times associated with obstetric consultants and radiology performed imaging. Prior literature shows significantly reduced emergency department length of stay when pelvic ultrasound was performed by emergency medicine physicians [17]. Other factors that can influence whether a POCUS examination is performed or not include the availability of radiology to perform an ultrasound, lack of confidence in obtaining or interpreting images by a provider, or lack of time to perform an exam in a busier practice environment [18,19].

These findings are similar to the data published by Smalley et al. in 2023 [10], who also found that cardiac (1,222), FAST (1,109), obstetric (826), and soft tissue (491) were the most commonly billed scans when evaluating 140 emergency physicians practicing in a community setting. Their study also found that ocular (179) and renal (127) POCUS exams were commonly performed, which reflected the practice by faculty in our study practicing in an academic setting. Ocular and renal exams were uncommonly performed when only evaluating faculty in our study practicing in a community setting. Lung exams were more commonly utilized in Smalley et al.'s study, while in our data, lung POCUS was mainly used by ultrasound division members and only moderately used by faculty in an academic setting without accounting for ultrasound division members. Lung POCUS was rarely used in the community setting. This difference in usage is likely a reflection of the difference in POCUS training of the operators and comfort with using lung POCUS between the two study settings and may suggest that faculty in our system, particularly those practicing in the community setting, may benefit from additional training with lung POCUS. 

The findings from this study, which included a 10-hospital healthcare system, along with those previously published by Smalley et al. [10], suggest that cardiac, FAST, obstetric, and soft tissue POCUS are the most commonly utilized POCUS examinations in the academic and community clinical setting. This data should serve as an initial starting point for adjusting POCUS guidelines focused on training and curricula development to be rooted in evidence instead of solely expert opinion.

Limitations

There are several limitations to consider. Data were obtained from a single healthcare system, which may introduce selection bias, limiting the generalizability of our results. Despite this limitation, the healthcare system is large, including 10 emergency department sites with 64 different faculty performing and billing for clinically indicated POCUS scans, and included data from academic, pediatric, and community sites. In addition, we evaluated utilization with and without faculty from the ultrasound division to be more representative of departments that have either no or a few POCUS fellowship/advanced trained faculty. Overall, the number of billed POCUS examinations in the pediatric (334) and community (524) settings was low. Lower usage in these practice settings is likely due to slower uptake of POCUS in the pediatric setting, lack of comfort with using POCUS to guide clinical care, and availability of radiology imaging. This study was also limited in that we did not evaluate procedural ultrasound. As previously stated, documentation of procedural POCUS was low in QPath with billing data being captured through the electronic medical record. Our QPath data would severely underrepresent the number of procedural POCUS exams being performed. This is a great area for further research. Lastly, it is more than likely that the number of POCUS exams performed during the study period was higher but that images were not recorded or submitted for billing. Physicians unfamiliar with the POCUS workflow may have had artificially depressed scan numbers as well, and in some instances, the workflow solution did not function properly to allow for billing. While those studies would not have been captured in this analysis, we believe that they represent a small subset of clinically indicated scans and are unlikely to impact the overall results from this dataset.

## Conclusions

Our data suggest that emergency physician POCUS curricula for resident and post-residency physicians should focus on the most clinically utilized POCUS exams, which in this dataset included cardiac, FAST, obstetric, and soft tissue exams. Lung, gallbladder, renal, and ocular were moderately used. Other diagnostic exams such as the aorta, DVT, testicular, and appendix, are still important from a clinical perspective to know how to perform and interpret, which we found to be rarely utilized in the clinical setting. Triaging educational resources towards these more commonly used scans may be a more practical use of resources and may ultimately lead to high utilization by faculty in the clinical setting. More data are needed from the pediatric setting to determine which scans are most utilized.
